# Control of White Spot Lesion Adjacent to Orthodontic Bracket with Use of Fluoride Varnish or Chlorhexidine Gel

**DOI:** 10.1155/2015/218452

**Published:** 2015-04-20

**Authors:** Manuel Restrepo, Diego G. Bussaneli, Fabiano Jeremias, Rita C. L. Cordeiro, Ana C. Magalhães, Denise M. Palomari Spolidorio, Lourdes Santos-Pinto

**Affiliations:** ^1^Department of Orthodontics and Pediatric Dentistry, Araraquara School of Dentistry, Universidade Estadual Paulista (Unesp), Rua Humaitá 1680, 14801-903 Araraquara, SP, Brazil; ^2^Department of Biological Sciences, Bauru School of Dentistry, University of São Paulo (USP), Alameda Octávio Pinheiro Brisolla 9-75, 17012-191 Bauru, SP, Brazil; ^3^Department of Physiology and Pathology, Araraquara School of Dentistry, Universidade Estadual Paulista (Unesp), Rua Humaitá 1680, 14801-903 Araraquara, SP, Brazil

## Abstract

The aims of this study were to compare the effectiveness of fluoride varnish and chlorhexidine gel in controlling white spot lesions (WSLs) adjacent to orthodontic brackets and to compare the ability of Quantitative Light-Induced Fluorescence (QLF) to measure mineral uptake with that of transverse microradiography (TMR). Thirty premolars with artificially induced WSLs were randomly assigned to three groups: (1) two applications of 5% NaF-varnish (*F*), with one-week interval, (2) two applications of 2% chlorhexidine gel (CHX), with one-week interval, and (3) control (CO), no treatment. QLF was used to measure changes in fluorescence before and after caries induction, 1 week after each application and 1, 2, and 3 months after the last application of *F* or CHX. TMR was performed to quantify lesion depth and mineral content after caries induction to evaluate the effects of *F*, CHX, and CO 3 months after the last application of agents. The data were analyzed by repeated measures ANOVA and Tukey's test. All treatments increased the mineral content during the experimental period; however, *F* induced faster remineralization than CHX. The correlation between QLF and TMR was significantly moderate. Two applications of fluoride varnish or 2% chlorhexidine gel at one-week intervals were effective in controlling WSLs.

## 1. Introduction

Enamel demineralization and gingivitis are particularly common problems during orthodontic treatment, and their treatment is one of the greatest challenges faced by clinicians. The presence of fixed appliances on tooth surfaces with brackets and bands makes it difficult to clean teeth, favor dental biofilm accumulation, in addition to increasing the prevalence of cariogenic and periodontopathogenic bacteria [1]. Clinically, the demineralization sites are detected as opaque and porous white spot lesions (WSLs) that may compromise the final result of the treatment. The early detection of WSLs adjacent to orthodontic brackets is important, in order to implement proper and noninvasive management, because, at this stage, lesions have the potential to be remineralized and can be monitored over time [2].

Conservative approaches to the management of WSLs using remineralizing therapies have become a subject of growing interest among clinicians and researchers. Among the available strategies, the use of fluorides has been shown to be highly effective in controlling caries lesions [3]. There is a body of scientific evidence that proves the benefits of fluoridated varnish in reducing the incidence of WSLs during orthodontic treatment [4]. The ease of application, safety, increase in contact time with enamel, and acceptance by patients have made this product one of the main choices for the management of WSLs [5]. Apart from the questionable necessity of professional applications of high concentrations of fluoride in active WSLs, the appropriate intervals for fluoride varnish in orthodontics patients remains undetermined. There is a very clear need for research on the best regimen to assist in the remineralization of early carious lesions [6].

Whereas other strategies have been adopted for biofilm control by the use of antimicrobials agents [7], chlorhexidine continues to be the most effective antimicrobial agent for the control of periodontal pathologies in the orthodontic patient [8]. However, the evidence regarding the effectiveness of chlorhexidine in controlling initial caries lesions is inconclusive [9].

It is well known that chlorhexidine inhibits acid production in biofilm and thus reduces the fall in pH during sucrose challenges [10]. Some authors have affirmed that one of the ways to paralyze initial lesions in enamel is to protect the body of the lesion from microorganisms, by the application of antimicrobial agents [11]. Therefore, more in vitro and in vivo studies on this field are needed to confirm this finding.

Conventional methods for caries detection (visual and radiographic examination) are not capable of quantifying the mineral loss or gain occurring as a result of demineralization and remineralization processes, respectively [12]. In this context, quantitative methods have been developed for caries detection and for monitoring clinical changes in the mineral content. Quantitative Light-Induced Fluorescence (QLF) is a validated method for assessment and longitudinal monitoring of mineral changes in the early stages of caries [13].

Therefore, the aims of this in vitro study were (1) to compare the effectiveness of fluoride varnish and 2% chlorhexidine gel for controlling WSLs adjacent to orthodontic brackets by using QLF and (2) to correlate the data obtained by QLF with those of transverse microradiography (TMR).

## 2. Materials and Methods

### 2.1. Sample

The sample consisted of 40 healthy human premolars, free of spots, cracks, and fractures. The teeth were freshly extracted for orthodontic reasons and donated, with the approval of the local Research Ethics Committee (Araraquara Dental School, Universidade Estadual Paulista, Unesp, process 29/11). Upon collection, the teeth were frozen at −20°C and stored at 100% relative humidity. Sample size calculations were based on detecting a difference of 30% reduction in QLF reading between the test group and the control group with a significance level of 5% with an 80% power.

### 2.2. Tooth Preparation

Before experimental use, the enamel surfaces were polished with nonfluoridated pumice and water slurry, rinsed with deionized water and dried with compressed air.

To standardize and limit the enamel area exposed to the etching and bonding procedures, the enamel surface was protected with dental wax during all adhesive procedures. Using a hole puncher, a window was cut from the modeling wax, leaving an enamel area corresponding to the orthodontic bracket base [14]. The enamel area was conditioned with 35% phosphoric acid (Unitek Etching Gel, 3M, Monrovia, USA) for 30 seconds and then thoroughly washed and dried. Transbond XT (3M, Monrovia, USA) was applied on the etched enamel and light-polymerized for 20 seconds. Brackets (Mini Diamond VS, Ormco, Orange, California, USA) were then placed 2 mm gingivally to the buccal cusp tip and in the mesiodistal center of the clinical crown and bonded with Transbond XT adhesive resin. After using a dental scaler to remove any residual adhesive around brackets, the resin was light-polymerized for 40 seconds (Elipar Freelight, 3M, Seefeld, Bavaria, Germany). Afterward, the dental wax was removed from each tooth.

**Figure 1:**
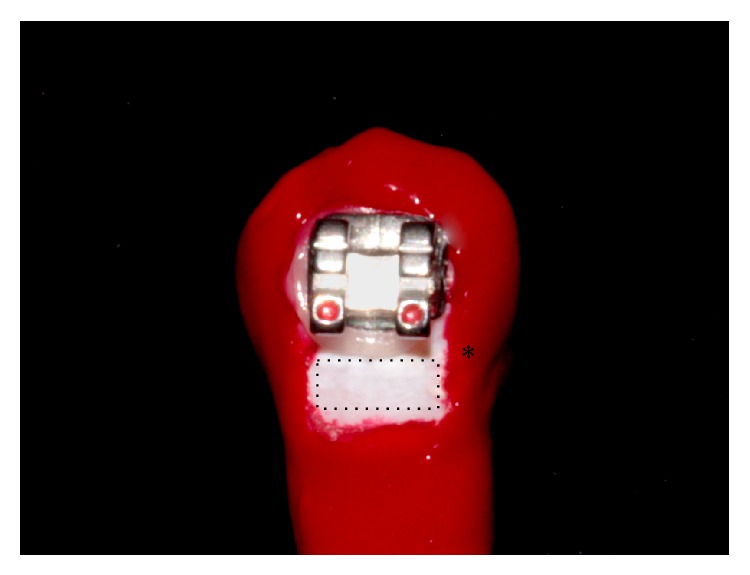
Photo showing a tooth with an orthodontic bracket and the enamel area that was exposed to the artificial demineralization (dotted rectangle). The asterisk (∗) indicate the control area for QLF measurements, covered with nail varnish.

The crowns and roots of the teeth were sealed with two coasts of acid resistant enamel (Colorama, Ceil, Com Exp Ind Ltda, São Paulo, SP, Brazil), leaving only a rectangular area measuring 2.5 mm × 2 mm exposed in the cervical region of the bracket for induction of the artificial demineralization process [14] (Figure 1).

### 2.3. Microbiological Caries Induction

The teeth were immersed in a cariogenic solution (pH around 4.0) containing 3.7 g of brain heart infusion culture supplemented with 0.5 g of yeast extract (Becton Dikinson and Company), 1.0 g of glucose (Synth; LabSynth, São Paulo, SP, Brazil), and 2.0 g of sucrose (Synth; LabSynth) per 100 mL distilled water. This solution was autoclaved for 20 min at 121°C and inoculated with young primary culture of* Streptococcus mutans* (ATCC 25175; Tropical Culture Collection, Andre Tosello Research Foundation, Campinas, SP, Brazil). The teeth were incubated in a microaerophilic environment at 37°C in a candle jar (BBL GasPak system, Becton-Dickinson, Franklin Lakes, USA) for 9 days. Every 48 hours, the teeth were transferred to another beaker containing a new artificial caries solution without inoculation of new microorganisms [15–17].

The biofilm formed on tooth surfaces was removed with gauze and the nail varnish was removed manually with a scalpel blade. The teeth were copiously washed in deionized water, revealing a white spot lesion adjacent to orthodontic bracket.

When the microbiological caries induction had been completed, 10 teeth were prepared for microradiographic analysis, which served as the gold standard for validation of mineral loss and lesion depth.

### 2.4. Groups

Thirty teeth were randomly allocated to three groups (*n* = 10). In the fluoride group (*F*) WSLs were treated with 5% NaF varnish (Duraphat, Colgate Palmolive, Hamburg, Germany). In the antimicrobial group (CHX), WSLs were treated with 2% chlorhexidine gel (Clorexal gel 2%, biodinâmica, Ibiporã, PR, Brazil). Both agents (*F* or CHX) were applied using a swab, two times with an interval of one week between applications. After 24 hours the remaining fluoride varnish or chlorhexidine was removed with a scalpel. In the control (CO) group, no professional treatment was performed. The teeth were only rubbed with cotton swabs imbibed with deionized water.

During the experiments the teeth were individually kept in 5 mL of artificial saliva (1.45 mM CaCl_2_·2H_2_O, 5.4 mM KH_2_PO_4_, 0.1 M Tris buffer, 2.2 g/l porcine gastric mucin, pH 7.0) at 37°C, which was changed every week.

### 2.5. QLF and TMR

The primary outcome was the change in fluorescence measured using a QLF (Inspektor Dental Care BV, Amsterdam, The Netherlands), at the following time intervals: before and after caries induction, 1 week after each application, and 1, 2, and 3 months after the last application of* F* or CHX.

The QLF measurements were performed in an environment with low light. The handpiece of the device was positioned parallel to the buccal surface. The image was captured and analyzed using the Inspektor Pro software program (version 2.0.0.32, Inspektor Dental Care BV, Amsterdam, The Netherlands) to delimit the WSL. The changes in fluorescence values were determined by the percentage difference between sound and demineralized areas of each test site. Δ*F* value was then recorded, considering a 5% limited level [18].

At the end of the experimental time and when the measurements with QLF had been completed (3 months later) the teeth were prepared for microradiographic analysis. The brackets were carefully debonded from the tooth surface with a bracket remover. The teeth were sectioned once with a diamond band saw, perpendicularly to the lesion to obtain tooth slices with a thickness of approximately 500 *μ*m. The tooth slices were then manually ground with water-cooled silicon carbide discs (600-, and 1200-grade papers; Buehler, Lake Bluff, USA) to a thickness of 80–100 *μ*m.

The slices were fixed in a sample-holder together with an aluminum calibration step wedge with 11 steps. A microradiograph was taken using an X-ray generator (Softex, Tokyo, Japan) on the glass plate at 20 kV and 20 mA (at a distance of 42 cm) for 20 min. The glass plates were developed for 5 min, rinsed in deionized water, fixed for 3 min in a dark environment, and then rinsed in running water for 10 min and air-dried (all procedures were performed at 20°C). The developed plate was analyzed using a transmitted light microscope fitted with a 20x objective (Zeiss, Germany), a CCD camera (Canon, Japan), and a computer. The images were taken using a data-acquisition software program (version 2012) and interpreted using calculation (version 2006) software programs from Inspektor Research System BV (Amsterdam, The Netherlands). Five parameters were obtained: the lesion depth (LD), the integrated mineral loss (Δ*Z*), the average mineral loss over the lesion depth (*R*), the mean thickness of the “pseudointact” surface layer (SL), and the maximum mineral content of the surface layer (*Z*_max⁡_).

### 2.6. Statistical Analysis

The data were analyzed using GraphPad Instat version 4.0 (San Diego, USA) and BioEstat version 5.0 (Tefé, AM, Brazil) software programs. The data set for each variable were evaluated concerning their distribution using the Kolmogorov-Smirnov test. The follow-up Δ*F* was analyzed using repeated-measures ANOVA and the post hoc Tukey test. The mineral loss (Δ*Z*) and lesion depth (LD) were compared using the Kruskal-Wallis test followed by Dunn's multiple comparison test and ANOVA, respectively. To analyze a possible relationship between Δ*F* and Δ*Z* the data were submitted to linear regression. The correlation between these measures was evaluated using Pearson's correlation coefficient. The level of significance for all tests was set at 5%.

## 3. Results

**Figure 2:**
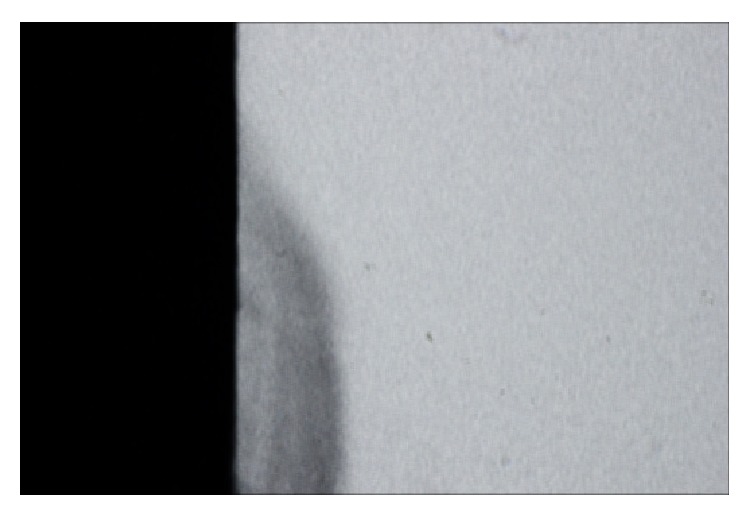
TMR image of a representative specimen after microbiological caries induction.

**Figure 3:**
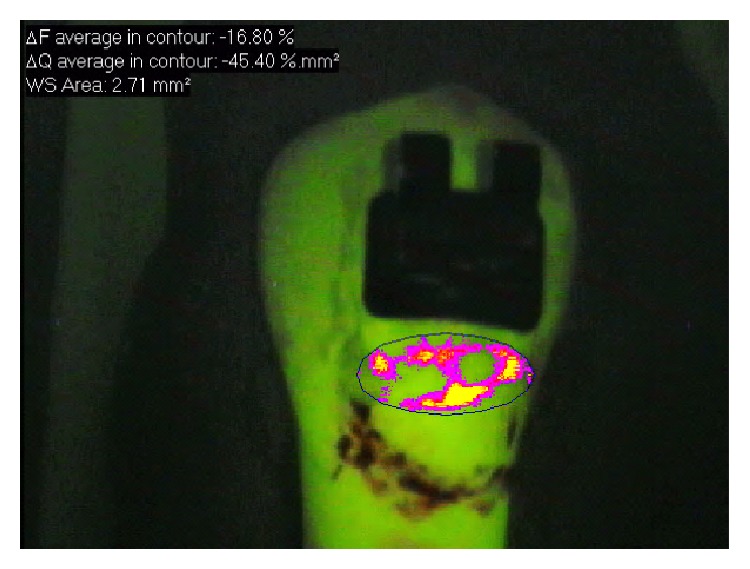
QLF image showing demineralized area adjacent to orthodontic bracket.

The microbiological caries induction model was able to produce a subsurface lesion (Figure 2) that was detected by the QLF (Figure 3).  Table 1 shows an overview of all TMR parameters after microbiological caries induction.

The results of the QLF measurements are summarized in Table 2. One week after the first application, only* F* significantly increased the fluorescence values, which were kept constant throughout the experimental period. Whereas, the increase in fluorescence values for CHX was observed 1 week after the second application, which was kept constant throughout the experimental period. It was only in the third month that the fluorescence values of the control group returned to being similar to those at baseline. When the groups were compared at baseline, after artificial WSL induction, and, at 3 months, no statistically significant differences were observed between them (*P* > 0.05).

Table 3 shows the comparison of the Δ*Z* (%vol/*μ*m) and lesion depth (*μ*m) values obtained after artificial WSL induction and after the third month. The Δ*Z* parameter values were statistically similar between Groups* F*, CHX, and control after 3 months, however, differing from the values presented at baseline. The lesion depth remained constant throughout the experimental period (Table 3) (*P* = 0.4212).

**Figure 4:**
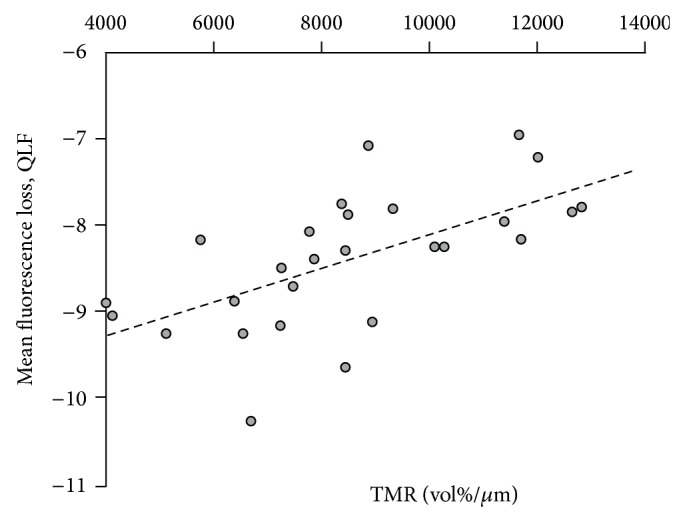
Correlation between QLF and TMR.

The Pearson correlation coefficient indicated moderate (*r* = 0.63, 95% CI = 0.35–0.81) but statistically significant positive correlation between Δ*Z* (TMR) and Δ*F* (QLF) (*P* = 0.0002) (Figure 4).

## 4. Discussion

Laboratory models have been widely applied in Cariology, allowing analysis of the different remineralizing treatments for WSLs [14, 19]. The microbial method is one of the protocols most used to induce caries lesion in dentin [15–17]. This was the first study to adapt this model to produce enamel WSL in permanent teeth. The main advantage of the microbial method in comparison with the abiotic types is the similarity to the clinical condition, considering the presence of biofilm and cariogenic challenges.

With respect to the diagnosis of dental caries, the conventional methods (visual and radiographic examination) present low sensitivity for quantifying the changes in mineral content as result of demineralization and remineralization [12]. To overcome this limitation, QLF has been studied as alternative method to quantify differences between sound and demineralized enamel, showing a correlation with TMR ranging from 0.62 to 0.84 for demineralized and from 0.66 to 0.84 for remineralized enamel [13, 20, 21]. In our study, we found a correlation coefficient of 0.63, in agreement with the literature. Therefore, QLF seems to be a valid method to quantify demineralization and monitor the treatment of WSLs [21, 22].

Orthodontic patients develop significantly more WSLs than nonorthodontic patients, which might compromise the final result of treatment. In addition, the progression of caries is faster in patients with full orthodontic appliances. WSLs can become noticeable around the brackets within 1 month after bracket placement, although the formation of regular caries usually takes at least 6 months [23]. Therefore, early diagnosis enables the clinician to implement minimally invasive treatments with the use of remineralizing therapies, with the goal of paralyzing lesion progression.

In our study, lesions treated with* F* varnish showed faster remineralization than the other treatments. Remineralization with the application of* F* varnish was stable throughout the period of 3 months. The benefit of this application regime could be the precipitation of CaF_2_-like layer on the enamel, thus increasing the remineralization of predemineralized enamel [24]. Our results support those of previous studies [6, 25] in which quantitative analyses were performed, and it was shown that if the frequency of professional fluoride application were increased, higher mineral contents could be obtained. It is important to note that the inhibition of enamel demineralization and the enhancement of remineralization are positively but not linearly related to the concentration of fluoride [25]. We do, however, believe that since brackets favor biofilm accumulation, they could also favor the retention of varnish, thereby increasing the contact time with enamel. Consequently the brackets would prolong the reactivity of NaF with the tooth surface [26] and justify its use in orthodontic patients with active WSLs.

The fact that CHX is an agent frequently indicated for chemical biofilm control in patients with orthodontic appliances, led us to evaluating its possible effect on the remineralization of active white spot lesions. Our results showed that two applications of 2% CHX gel increased the fluorescence values. These results cannot be attributed exclusively to the antimicrobial property of CHX, which allows us to hypothesize that its effect on the remineralization of WSL may be due to electrostatic links with the phosphate groups present in the hydroxyapatite of the dental structure and artificial saliva, which could favor the precipitation of phosphate salts on the reactive surface of demineralized enamel [27].

Although the results of this in vitro study were positive, the clinical situation is different, because remineralization without treatment (control group) is seldom achieved, particularly when we consider the high risk of the orthodontic patient and the microbiological dynamics of the oral environment. We believe that the absence of cariogenic biofilm and carbohydrates, in addition to the presence of conditions favorable to remineralization (storage of teeth in artificial saliva containing Ca and P ions) may have had an influence on the increase in mineral content in the control group.

Further studies, using models more close to the in vivo condition, are necessary to understand the action of CHX. Furthermore, it is important to evaluate the effect of frequency of application of* F* or CHX, associated (or not) with oral hygiene instruction, on the remineralization of WSL in vivo, to establish a better clinical protocol, showing efficiency and a good cost-benefit for orthodontic patients with active WSL.

## 5. Conclusion

Two applications of fluoride varnish or 2% chlorhexidine gel with a one-week interval were effective in controlling WSLs adjacent to orthodontic brackets. However, the* F* varnish showed a faster action, which might be an advantage in the clinical condition. QLF was effective in detecting demineralization and remineralization adjacent to orthodontic brackets.

## Figures and Tables

**Table 1:** Summary for all TMR parameters (mean ± SD).

Mineral loss	Lesion depth	Ratio	Lesion width	Thickness	SS layer	Lesion body
(Vol%, *μ*m)	(*μ*m)	(Vol%)	(*μ*m)	(*μ*m)	(*μ*m, Vol%)	(*μ*m, Vol%)
10174.4 ± 2060.3	248.3 ± 58.4	42.2 ± 9.24	18.3 ± 6.6	42.8 ± 5.9	88.0 ± 44.6	33.3 ± 5.6

*n* = 10, after microbiological caries induction.

**Table 2:** Mean and SD of the fluorescence values for each group during the experimental time.

Group	Baseline	After artificial induction of WSL	1 week after the 1st application	1 week after the 2nd application	1 month	2 months	3 months
*F*	−7.04 ± 0.83^aA^	−13.03 ± 3.77^aB^	−8.77 ± 2.01^aA^	−6.92 ± 0.52^aA^	−7.82 ± 2.25^aA^	−7.60 ± 1.88^aA^	−8.03 ± 1.89^aA^
CHX gel	−7.32 ± 0.96^aA^	−13.84 ± 5.24^aB^	−11.42 ± 4.77^aB^	−8.05 ± 1.69^abA^	−8.34 ± 1.38^aA^	−8.25 ± 0.99^aA^	−8.10 ± 0.94^Aa^
Control	−6.84 ± 1.47^aA^	−12.42 ± 2.45^aB^	−10.98 ± 3.89^aB^	−11.28 ± 3.19^bB^	−10.12 ± 1.78^aB^	−10.20 ± 1.73^aB^	−8.58 ± 0.063^aA^

Different lower case letters within the same column show significant differences among the treatments. Different capital letters within the same row show significant differences among the periods of remineralization (repeated-measures ANOVA and Tukey's tests).

**Table 3:** Summary and statistical comparison for TMR parameters after WSL induction and 3 months after the last application of *F* or CHX (mean ± SD).

TMR parameter	After artificial induction of WSL	*F* ^*^	CHX^*^	Control^*^
Δ*Z* (%vol/*μ*m)	10174.4 ± 2060.3^a^	7459 ± 960.1^b^	7670 ± 7699.6^b^	7608 ± 7608^b^
Lesion depth (*μ*m)	248.3 ± 58.5^A^	224.43 ± 76.3^A^	266.7 ± 87.2^A^	208.9 ± 92.8^A^

Different superscript letters in the same line show significant difference among the groups (Kruskal-Wallis for Δ*Z* and ANOVA for lesion depth).

^*^Performed at the end of the experimental time interval, 3 months after the last application of *F* or CHX.
